# The effect of neural mobilisation on cervico-brachial pain: design of a randomised controlled trial

**DOI:** 10.1186/1471-2474-15-419

**Published:** 2014-12-10

**Authors:** Cato A Basson, Aimee Stewart, Witness Mudzi

**Affiliations:** Private practice, 407 Stonewall Avenue, Faerie Glen 0043, PO Box 74745, Lynnwood Ridge, 0040 South Africa; Physiotherapy Department, Faculty of Health Sciences, University of the Witwatersrand, 7 York Road, Park Town, 2193 Johannesburg South Africa

**Keywords:** Neck pain, Cervico-brachial pain, Neural mobilisation, Neuropathic pain, Catastrophising

## Abstract

**Background:**

Neck pain is a common musculoskeletal complaint and is often associated with shoulder or arm pain. There is a paucity of information on effective treatment for neck and arm pain, such as radiculopathy or cervico-brachial pain. Guidelines recommend neck mobilisation/ manipulation, exercises and advice as the treatment for neck pain, and neck and arm pain. There are a few studies that have used neural mobilisation as the treatment for cervico-brachial pain. Although results seem promising the studies have small sample sizes that make it difficult to draw definite conclusions.

**Methods:**

A randomised controlled trial will be used to establish the effect of neural mobilisation on the pain, function and quality of life of patients with cervico-brachial pain. Patients will be recruited in four physiotherapy private practices and randomly assigned to usual care or usual care plus neural mobilisation.

**Discussion:**

In clinical practice neural mobilisations is commonly used for cervico-brachial pain. Although study outcomes seem promising, most studies have small participant numbers. Targeting the neural structures as part of the management plan for a subgroup of patients with nerve mechano-sensitivity seems feasible. Patients with neuropathic pain and psychosocial risk factors such as catastrophising, respond poorly to treatment. Although a recent study found these patients less likely to respond to neural mobilisation, the current study will be able to assess whether neural mobilisation has any added benefit compared to usual care. The study will contribute to the knowledge base of treatment of patients with cervico-brachial pain. The findings of the study will be published in an appropriate journal.

**Trial registration:**

Trial registration Number: PACTR201303000500157.

**Electronic supplementary material:**

The online version of this article (doi:10.1186/1471-2474-15-419) contains supplementary material, which is available to authorized users.

## Background

Neck pain is one of the most common debilitating musculoskeletal complaints seen in physiotherapy practice [1–3]. In a systematic review on the prevalence of neck pain in the adult population, Fejer et al. found the point prevalence to be 7.6% and the lifetime prevalence to be 48.6% [4]. In the 2010 global burden of disease report neck pain ranked as the fourth highest disease in terms of disability [5].

Neck pain is often associated with headache, upper back and shoulder/arm pain [3, 6]. In a study by Daffner et al. [7] 65.4% of the neck pain population included in their study had arm pain associated with their neck pain. The patients with neck and arm pain were more disabled than patients with only neck pain. Cervico-brachial pain syndrome is an upper quarter pain syndrome in which neural tissue sensitivity to mechanical stimulus is thought to play a role [8, 9]. Different terms are often used to describe patients with upper quadrant pain such as cervico-brachial pain [10], nerve related neck and arm pain [11] and cervical radiculopathy [6, 12]. Diagnosis of cervico-brachial pain is made by a clinical process and there is often no overt neural involvement [8]. Neural involvement can only be assumed if a cluster of clinical findings are present such as an active and passive movement dysfunction, adverse response to neurodynamic testing and evidence of a local cause of neuropathic pain [8, 9].

Neuropathic pain is described as pain initiated or caused by a primary lesion or dysfunction in the nervous system [13]. Neuropathic pain is a problem associated with and prevalent in many musculoskeletal conditions such as low back pain [14], acute or chronic radiculopathy [12] and syndromes such as cervico-brachial pain syndrome [10, 15]. Neuropathic pain is consistently linked to high levels of pain, disability, poor quality of life and poor response to treatment [14, 16, 17] and is therefore a difficult condition to treat successfully [14, 16].

Psychosocial factors have also been shown to play an important role in treatment outcomes [18–21]. Pool et al. [19] examined the influence of different psychosocial factors on treatment outcome and found fear of movement to be significantly correlated with poor outcomes in the short and long term. In a study by Karels et al. catastrophising was significantly linked to persistent symptoms of neck pain over a six-month period [22]. Catastrophising is a cognitive process that includes elements of magnification, helplessness, pessimism and is a consistently important predictor of poor pain-related outcomes [23]. Verhagen et al. [20] explored the influence of various factors on treatment outcome in neck pain and found pain severity and catastrophising to be the most important determinants for poor recovery. In a study to examine factors influencing return to work in patients with neck/arm/shoulder pain, the authors also concluded that psychosocial factors should be taken into account when interventions are planned [18]. Similar to these findings Thompson et al. [24] found catastrophising and poor self-efficacy to be associated with higher pain and disability. Other factors that have been shown to influence treatment outcomes are pain intensity at baseline, increased age and the presence of low back pain [25, 26].

Most clinical guidelines do not differentiate between treatment for neck pain and neck and arm pain such as cervico-brachial pain [27]. The American Physical Therapy Association recommends mobilisation/ manipulation of the neck and thoracic spine, exercises and education in the management of neck pain [27]. The findings of a recent Cochrane review confirm the above recommendations [28]. The American guidelines [27] also recommend that neural mobilisation should be considered for patients with neck and arm pain (level B evidence).

Neural mobilisations (NM) are often used to affect the neural structures in conditions with signs of neural involvement or neural mechano-sensitivity [10, 11]. NM is said to affect the axoplasmic flow [29], movement of the nerve and its connective tissue [30], and the circulation of the nerve by alteration of the pressure in the nervous system and dispersion of intraneural oedema [31]. NM can also decrease the excitability of dorsal horn cells [32]. NM is defined as interventions aimed at affecting the neural structures or surrounding tissue (interface) directly or indirectly through manual techniques or exercises. The interface can be mobilised by mobilising the tissue surrounding the nerve, along the course of the nerve [33]. Although it is a technique used in clinical practice the use thereof in the literature could only be identified in a case report of a patient with cervical radiculopathy [34]. The treatment was combined with manual therapy and exercises, both treatments which have been shown to be effective for cervical radiculopathy [28]. It is therefore difficult to know whether the NM contributed to the treatment effect. The effectiveness of this form of NM has therefore yet to be established. In cervico-brachial pain, neural tissue sensitivity to mechanical stimulus is thought to play a role [8], it can therefore be reasoned that targeting the neural structures specifically should be an important aim of treatment in patients with cervico-brachial pain.

The efficacy of NM has been studied in various populations such as low back pain [35], carpal tunnel syndrome [36], lateral epicondalalgia [37] and cervico-brachial pain [10, 11, 15]. The NM techniques used for cervico-brachial pain consisted of cervical lateral glides and neural gliding exercises [10, 15, 38]. According to a systematic review of NM, the evidence for the efficacy of NM is limited [39].

### Aims of study

The aims of the study are firstly to establish the effect of NM on the pain, function and quality of life of patients with acute and sub-acute cervico-brachial pain. Secondly it aims to establish if high catastrophising scores and neuropathic pain have an influence on treatment outcomes.

### Study design

A randomised controlled trial will be used to answer the research questions. The study will comprise of two groups, a control group and an experimental group. The control group will receive “usual care” (UC) [40] as identified by a review of the literature. The intervention group will receive neural mobilisation in addition to the usual care.

### Subjects

#### Selection procedure

**Figure 1:**
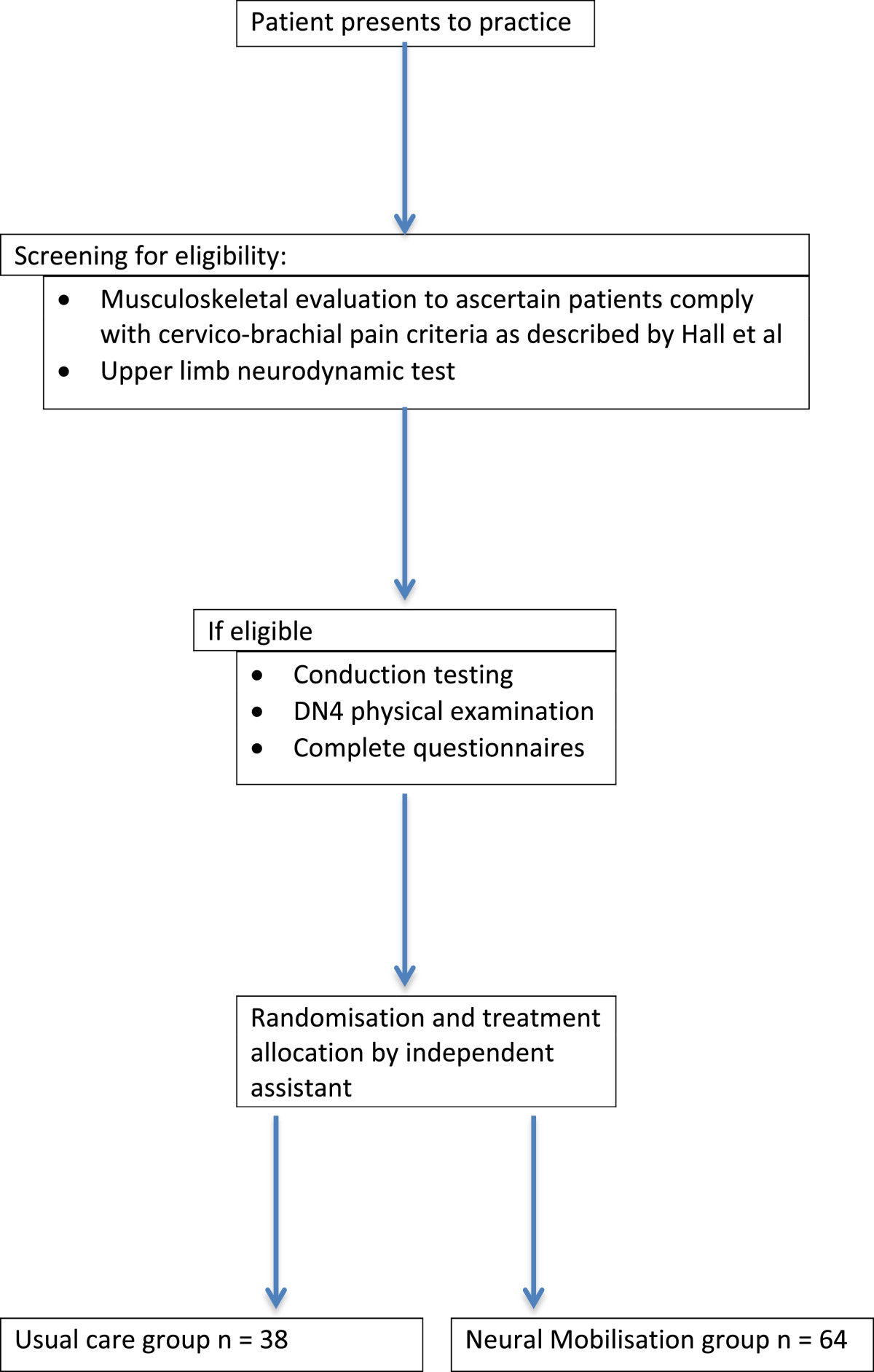
**Screening and allocation of patients.**

Patients will be recruited from private physiotherapy practices, which have agreed to take part in the study. These practices will be situated in the Pretoria region, Gauteng, South Africa. Patients are referred to private practices by general practitioners or may be self-referred. Patients presenting with cervico-brachial pain will be screened for eligibility and if eligible will be invited to take part in the study (Figure 1).

To be included in the study patients must be over the age of 18, with cervico-brachial pain as defined by Hall and Elvey [8]. This includes at least five of the following characteristics; antalgic posture, active movement dysfunction, passive movement dysfunction which correlate with the active movement dysfunction, adverse responses to neurodynamic tests which must relate specifically and anatomically to the active and passive movement dysfunction, and mechanical allodynia in response to palpation of specific nerve trunks. Lastly evidence from the physical examination of a local cause of the neurogenic pain must be present [8]. The upper limb neurodynamic test 1 (ULNDT1) will be considered positive if the patient’s pain is reproduced or partially reproduced by the test and changed by structural differentiation [41]. Patients with pain from recent onset (one day) up to 12 weeks, recurrent or first incident will be considered for inclusion. Patients with bilateral arm pain will also be considered for inclusion if they comply with the other inclusion criteria. Pain due to injury and the duration of pain will be recorded in the demographics questionnaire that patients will complete.

Patients who do not have a positive neurodynamic test will not be included in the study. Patients will be excluded from the study if they have had previous surgery or recent fractures of the cervical spine, serious neurological signs such as signs of spinal cord pressure or involvement of more than two nerve roots, conditions with long tract signs and those caused by other pathological diseases such as rheumatoid arthritis, neurological diseases, stroke, cerebral palsy, carcinoma or other red flags.

### Sample size

Primarily this study sets out to assess whether, 6 weeks after onset of treatment the Numerical Pain Rating Scale NPRS score in the intervention group is reduced compared to the UC group. A blocked 2:1 ratio randomisation for intervention to usual care will be done using a computer generated random number list. A sample size of 68:34 i.e. a total sample of 102 patients will have 90% power to detect a clinically relevant increase of 2 for the change from baseline at six weeks in the NPRS score. A standard deviation of 2.05 was assumed as derived from the effect size reported by Bolton and Wilkinson [42]. The standard deviation was furthermore inflated by √2 since change from baseline is of interest. A dropout rate of 15 – 20% was assumed. One-sided testing will be done at the 0.05 level of significance. (nQuery advisor 7).

### Ethical considerations

Written consent to take part in the study will be obtained from all study participants and they will receive an information sheet explaining the study. Written consent will be obtained from treating physiotherapists. Ethical approval for the study was obtained from the Human Research Ethics Committee of the University of the Witwatersrand, Johannesburg, South Africa.

### Outcome measures

The treating physiotherapist will administer the first set of questionnaires. Treatment randomisation will only be done as described above, after initial measures have been taken. All subsequent follow up questionnaires will be administered by a research assistant who will be blinded to group allocation as the follow up questionnaires at three weeks and six weeks will be done telephonically. The primary outcomes are pain (Numeric Pain Rating Scale), function (Patient Specific Functional Scale) and quality of life (EuroQual Instrument). These self-report outcomes will be followed up at three weeks, six weeks, six months and 12 months.

The NPRS will be used to measure the patient’s pain. The NPRS is an 11-point scale where patients are asked to rate their pain as 0 representing “no pain” and 10 “worst pain possible”. In a study comparing three pain measures [42]. Bolton and Wilkinson [42] found the NPRS to be the most responsive pain measure with an effect size of 0.86 [42]. The validity of the NPRS in an acute setting was established by Bijur et al. [43] and is strongly correlated with the visual analogue scale (r = 0.94, 95% Confidence Interval 0.93 – 0.95) [43]. The NPRS is as sensitive to change as the visual analogue scale which is currently considered the “gold standard” [44].

The Patient Specific Functional Scale (PSFS) will be used to measure disability. The PSFS is a self-report measure to rate activity limitation and function. Patients are asked to nominate three to five activities that are difficult to perform and rate them on an 11 point scale where 0 equals “unable to perform activity” and 10 represents “able to perform activity as before”. The scale was initially developed for patients with back pain and validated for patients with neck pain by Westaway et al. [45]. The scale has excellent validity (r = 0.73 - 0.83) when compared to the Neck Disability Index [45]. The test retest reliability coefficient is excellent (ICC = 0.97) [46]. Cleland et al. [47] compared the Neck Disability Index (NDI) and the PSFS in patients with cervical radiculopathy and found that the PSFS was more responsive than the NDI in this population [47]. In their study the test-retest reliability coefficient was good (ICC 0.82). The scale is commonly used in physiotherapy practice [48].

EuroQuol Instrument (EQ-5D) is a quality of life measurement and will be used to rate the quality of life of study participants. It has two sections: the first part consists of five sections namely mobility, self-care, usual activities, pain/discomfort and anxiety/depression. Each section is rated by three descriptions from “I have no problem” to “I am unable”. The second section of the questionnaire has a 20 cm Visual Analogue Scale with “best imaginable health state” at the one end and “worst imaginable health state” at the other end. The validity was established by measuring it against the SF-12 and positive correlations were found (r = 0.41). It has excellent reliability (ICC 0.82) [49]. Peolsson et al. [50] measured quality of life using the EQ5D in patients with cervical radiculopathy and whiplash associated disorder and found health related quality of life worse in these populations compared to a healthy group.

Patients will be asked to complete the Global Rating of Change Scale (GROC) six weeks after treatment has commenced. This scale measures the patient’s impression of improvement after an intervention. According to a review there are eight different scales [51]. In this study an 11 point Likert scale will be used with −5 representing very much worse, 0 is unchanged and +5 is fully recovered [51]. The test-retest reliability coefficient is good (ICC of 0.9). The scale correlates significantly with changes on the Roland Morris, Oswestry, Pain Rating Scale and the EQ-5D. According to Kamper et al. the Minimal Clinically Important Change of the GROC is two points on an 11 point scale [51]. Patients with a score of +3 or more will be considered “responders” and those with a lower score as “non-responders”.

Two questionnaires will be used to establish which patients have neuropathic pain and which patients are catastrophisers. These questionnaires will be repeated at six months and 12 months to measure if any change has taken place from initial assessment to one year follow up.

The Neuropathic Pain Diagnostic questionnaire (DN4) consists of two sections namely an interview and examination. The interview has two questions, the first about the characteristics of the pain (e.g. burning) and the second about associated symptoms (such as pins and needles) to which the answer is either yes or no. In the examination, tests for hypoesthesia to touch and prick in the painful area as well as whether or not brushing aggravates the pain, are done. Each positive scores a point with a total score of 10. A patient with a score of 4/10 or more can be diagnosed with neuropathic pain [40]. The sensitivity of the test at the cutoff of four is 82.9 and the specificity is 89.9. The inter-rater reliability has Kappa values of between 0.70 and 0.96 [40]. This test have been used successfully in a South African population that are not all English first language speakers [52]. The questionnaire is recommended for use in the South African Guidelines for management of neuropathic pain [53].

The Pain Catastrophising Scale (PCS) is a questionnaire that establishes the levels of catastrophising present in patients. The questionnaire has three components: magnification, rumination and helplessness. Participants are asked to reflect on past painful experiences and to indicate the degree to which they experienced each of 13 thoughts or feelings when experiencing pain. It is scored on a 5-point scale from 0 “not at all” to 4 “all the time” with a maximum score of 52. A score above 24 classifies the patient as a catastrophiser [23]. The internal consistency of the three subscales are Crohnbach’s α of Rumination α = 0.85, Magnification α = 0.75 and Helplessness α = 0.85 [54]. The reliability as tested by Osman et al. [54] could correctly identify 77.1% of the cases. Osman et al. [54] also confirm the findings of Sullivan et al. [23] that the three dimensions represent a single construct.

The Upper Limb Neurodynamic Test 1 (ULNDT1) is described as the straight leg raise test of the arm [33]. The upper limb nerves are elongated and moved in their nerve bed [55] as was verified in an in vivo study. The test consist of different components of movement: patient supine, neck in neutral, shoulder abduction to +/− 110°, extended wrist and fingers, forearm supination, shoulder lateral rotation and the amount of elbow extension is then measured [33]. The reliability of measuring onset of pain and sub-maximal pain in a clinical setting in patients with cervico-brachial pain was established as excellent by Coppieters et al. (ICC ≥ 0.98; SEM ≤ 3.4°) [56]. It is a valid way of identifying patients with a peripheral neuropathic pain component [41]. The ULNDT1 will be assessed again at six months and 12 months.

## Methods

### Pilot study

Four physiotherapists in private practice (including the researcher) will be the treating physiotherapists. All the physiotherapists are qualified manual therapists with knowledge of neural mobilisations. All participating physiotherapists will take part in a training workshop run by the researcher. The training workshop will consist of: training of the application of neural mobilisation along the course of the nerve, protocol for treatment groups, outcomes measures, baseline measures as well as patient screening.

The inter- and intra-rater reliability of the researcher and the research assistant was established for the ULNDT1. Inter-class correlation coefficient was good at 0.85, (95% CI 0.66-1.06). Intra-class correlation coefficient for physiotherapist 1: 0.85, (9 5% CI 0.64 -1.06) and for physiotherapist 2: 0.70 (95% CI 0.31- 1.09) which was acceptable.

### Initial assessment

Patients presenting at participating physiotherapy practices with neck and arm pain will be screened for inclusion into the study. Suitable candidates will receive a full musculoskeletal examination to confirm that they comply with the criteria for cervico-brachial pain as described above [8]. They will then complete the questionnaires as discussed.

Physical examination will include the ULNDT1. The test will be done as described above [33]. The range of ULNDT1 will be measured using a standard goniometer to measure the range of elbow extension [57]. The examination section of the DN4, that is; testing for hypoesthesia to touch, and prick and pain caused by brushing will be done at baseline, six months and 12 months. Neural conduction tests of sensation, muscle power and reflexes will be done according to Petty & Moore [58] at six and 12 months. They will also be asked to complete a demographic questionnaire on age, gender, duration of symptoms, previous neck pain, injury or insidious onset, education, occupation, sport, presence of headache or dizziness and indicate the area of pain on a body chart.

### Randomisation

Block Randomisation with a 2:1 ratio in blocks of 6 will be done [59, 60]. According to Moher et al. [61] an unequal randomisation is ideal for smaller randomised controlled trials and multicentre trials [61]. Randomisation will be done using a computer random generator [62]. An independent research assistant, naïve to study content, will be contacted by telephone. This research assistant will give a patient number sequentially and treatment (group) allocation to participating physiotherapists after all baseline measurements has been done.

Physiotherapists will receive an envelope with the patient information leaflet, consent form, treatment recording sheet and all questionnaires. Group allocation will not be visible and the research assistant doing follow up measurements will be blinded to group allocation. Treatment recording sheets will only be collected at the end of the data collection period. The treatment sheets will be used to assess whether treatment allocation was followed, number of treatment sessions and to screen for any adverse events due to treatment.

### Intervention

The American Physical Therapy Association Guidelines [27] as well as the Australian Guidelines [63] both recommend the use of a multimodal intervention comprising of gentle exercise, advice to stay active and cervical mobilisation/manipulation. The usual care (UC) [40] will be given according to these guidelines to ensure that all patients receive evidence based care. No specific mobilisation/ manipulation technique have been shown to be superior to another [64, 28].

The UC will consist of mobilisation of the cervical and thoracic spine, exercises and advice to stay active. As most physiotherapists in South Africa have been Maitland trained, the patients will receive Maitland mobilisation [65] of the cervical and thoracic spine. Exercises will include postural correction, deep neck flexor training according to Falla et al. [66] and strengthening and mobilising exercises using yellow Theraband™ as described by [67]. All study participants will receive advice to stay active [27]. The number of treatments will range between two and a maximum of eight treatments and will be recorded. Patients who have not improved by the sixth treatment or patients who report any adverse events will be referred back to their general practitioners. Patients in the UC group will not receive any neural mobilisations.

The intervention group will receive the NM in addition to UC. The NM used in this study will be done as described by Butler [33], a gentle soft tissue mobilisation of the neural container/interface “along the tract” ([33] pp. 380) of the nerve – directly where the nerve is palpable and indirectly where it lies deeper. The treatment will concentrate on areas where the nerve is mechano-sensitive to palpation and will be done from the hand or elbow (depending on patient’s area of pain) and followed up along the arm, first rib, scalene and into the neck. Mobilisation will first be done in a position where the nerve is relaxed, not provoking any of the patient’s symptoms. Palpation may only provoke minimal symptoms and should disappear as soon as it is stopped. The basic principles of neural mobilisations will be used to progress treatment; that is to commence treatment in the acute phase with the nerve in a neutral, non-tension position and to progress into a more tensioned position as pain and irritability improves [33, 68].

### Statistical considerations

Primarily this study sets out to assess whether, six weeks after onset of treatment the NPRS score in the intervention group is reduced compared to the UC group. The sample size calculation is reported for the NPRS, which require the largest sample size.

### Data analysis

The data summary will employ descriptive statistics, means, standard deviations and 95% confidence intervals and will be reported by study group (intervention & standard care) for the NPRS, PSFS and EQ-5D. Catastrophising (yes & no) and neuropathic pain (yes & no) will be summarised using frequencies, percentages, cross tables and 95% confidence intervals.

Two treatment groups will be compared with respect to the change from baseline at six weeks in the NPRS, PSFS and EQ-5D scores using an analysis of covariance (ANCOVA) with baseline scores, catastrophising (yes & no) and neuropathic pain (yes & no) as covariates. For the total assessment period of 12 months the interaction between visits and treatment will be assessed with respect to NPRS, PSFS and EQ-5D scores in a linear mixed model analysis. Testing will be done at the 0.05 level of significance. For all data description and analyses an intention-to-treat analysis will be performed.

## Discussion

Evidence for the treatment of neck and arm pain (including radiculopathy) is sparse [28]. In a recent review of non-invasive treatment for cervico-brachial pain, Salt et al. [6] found inconclusive evidence for the effectiveness of treatment on pain. Potential benefit was found for manual therapy, exercise and behavioural change approaches. The effect of treatment on function in this population group was mixed. They recommend that studies investigate which subgroups of patients will respond to specific interventions. It is postulated that mechano sensitivity of the nerves plays a role in cervico-brachial pain [8, 9] In this study the effect of neural mobilisation on a population with cervico-brachial pain and nerve mechano-sensitivity will be investigated. Clinical reasoning suggests that targeting the neural structures in these patients could be beneficial.

## Conclusion

The findings of this study should add to the knowledge base on the management of patients with cervico-brachial pain. Results of this study will be published in an appropriate journal.

## Authors’ original submitted files for images

Below are the links to the authors’ original submitted files for images. Authors’ original file for figure 1
