# Supplementary material for: The effect of neural mobilisation on cervico-brachial pain: design of a randomised controlled trial
Source: BMC Musculoskelet Disord. 2014 Dec 10;15:419. doi: 10.1186/1471-2474-15-419 (PMC4295331; doi:10.1186/1471-2474-15-419)
Supplement: Supplementary file 1 — Authors’ original file for figure 1 [file 12891_2013_2352_MOESM1_ESM.pdf]

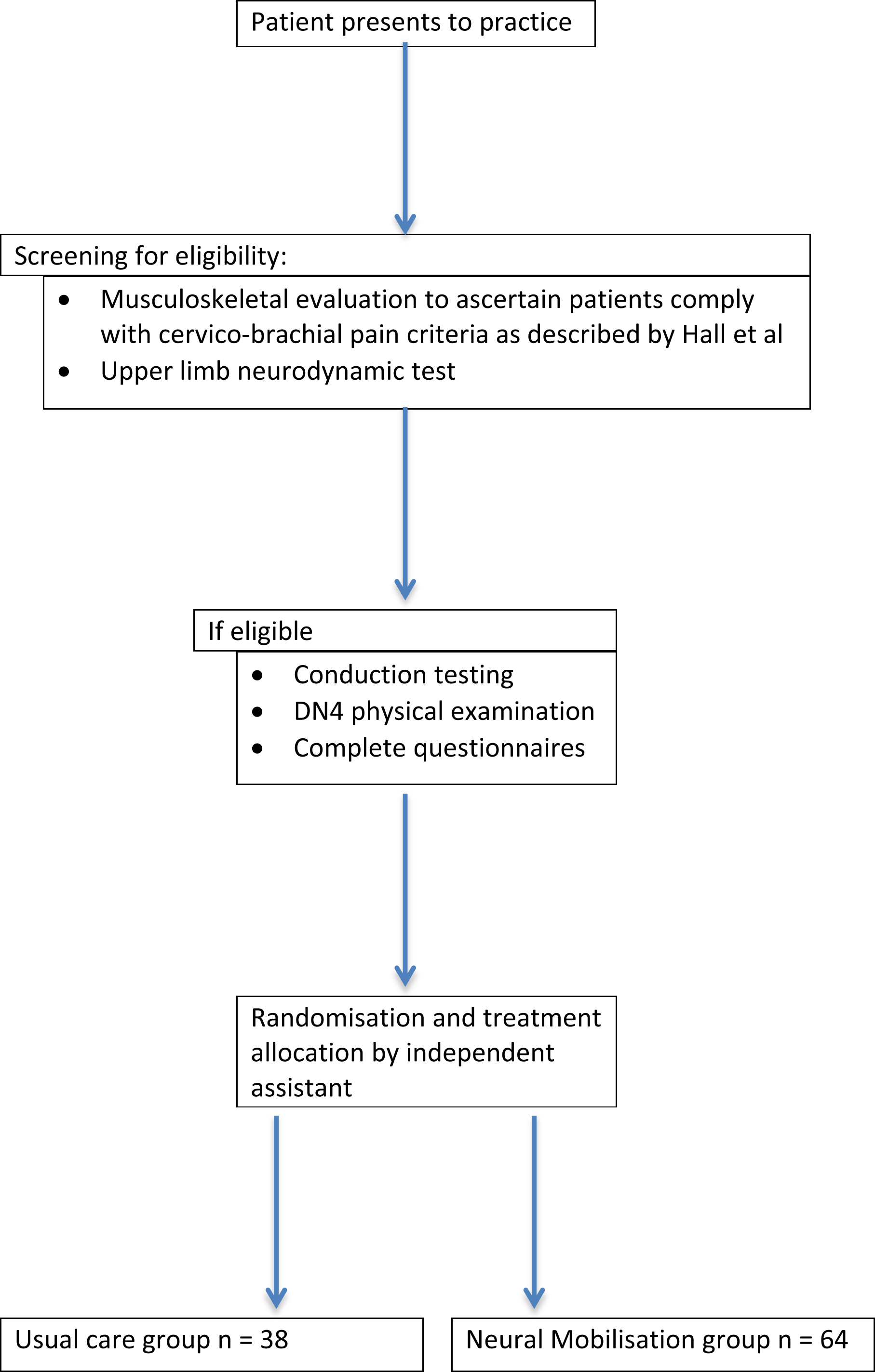
Patient presents to practice
Screening for eligibility:
• Musculoskeletal evaluation to ascertain patients comply with cervico-brachial pain criteria as described by Hall et al
• Upper limb neurodynamic test
If eligible
• Conduction testing
• DN4 physical examination
• Complete questionnaires
Randomisation and treatment allocation by independent assistant
Usual care group n = 38
Neural Mobilisation group n = 64
